# Correlation between blood cell indices and adiponectin and leptin levels in COVID-19

**DOI:** 10.17305/bb.2024.11153

**Published:** 2024-10-02

**Authors:** Mia Manojlovic, Branislava Ilincic, Marko Bojovic, Ivor Kolarski, Dragana Tomic Naglic, Edita Stokic, Sonja Zafirovic, Esma R Isenovic

**Affiliations:** 1 University of Novi Sad, Faculty of Medicine in Novi Sad, Novi Sad, Serbia; 2 Clinic for Endocrinology, Diabetes and Metabolic Disorders, University Clinical Center of Vojvodina, Novi Sad, Serbia; 3 Center of Laboratory Diagnostic, University Clinical Center of Vojvodina, Novi Sad, Serbia; 4 Clinic for Radiation Oncology, Oncology Institute of Vojvodina, Novi Sad, Serbia; 5 Center for Forensic Medicine, Toxicology and Molecular Genetics, University Clinical Center of Vojvodina, Novi Sad, Serbia; 6 Department of Radiobiology and Molecular Genetics, “VINČA” Institute of Nuclear Sciences - National Institute of the Republic of Serbia, University of Belgrade, Belgrade, Serbia

**Keywords:** Adiponectin, leptin, obesity, blood cell indices, platelets, COVID-19

## Abstract

Adipose tissue (AT) is a major metabolic organ, functioning through autocrine, paracrine, and endocrine mechanisms. This study investigated the relationship between adipokine levels and blood cell indices, particularly platelets, in individuals with COVID-19. Another aim was to enhance the understanding of AT’s endocrine function during dynamic pathological changes, such as acute viral infections like COVID-19. The study was conducted as a cross-sectional analysis at the University Clinical Center of Vojvodina in 2021 and 2022, including 76 consecutive SARS-CoV-2-positive patients of both sexes. Study parameters were determined from peripheral venous blood samples routinely collected upon hospital admission. The results showed that leptin levels were significantly positively correlated with body mass index (BMI) (ρ ═ 0.421, *P* < 0.001) and body fat mass (BFM) (ρ ═ 0.547, *P* < 0.001). Simultaneously, a significant negative correlation was observed between adiponectin levels and BMI (ρ ═ −0.430, *P* < 0.001). Additionally, a significant positive correlation was found between leptin levels and mean platelet volume (MPV) (ρ ═ 0.307, *P* < 0.05), platelet distribution width (PDW) (ρ ═ 0.325, *P* < 0.05), and platelet-large cell ratio (P-LCR) (ρ ═ 0.305, *P* < 0.05). Leptin’s impact on platelet indices was confirmed in both simple and multiple linear regression models, where leptin exhibited a slightly higher beta coefficient than BMI. In contrast, adiponectin levels were negatively correlated with hematocrit (HCT) (ρ ═ −0.329, *P* < 0.05). These findings may provide further insight into the previously suspected role of AT in the complex cascade of COVID-19 pathogenesis and platelet activation.

## Introduction

Adipose tissue (AT) is a major metabolic organ that plays a significant role through its autocrine, paracrine, and endocrine functions. AT secretes a wide array of adipokines and other adipose-derived factors, which are essential in intra- and inter-tissue signaling. The two most abundantly produced adipokines are adiponectin and leptin [1].

Adiponectin has several protective effects on various cell types, including increased insulin sensitivity, anti-inflammatory actions, antiproliferative effects, and protection against the development of atherosclerosis and carcinogenesis. Unlike other adipokines, adiponectin is thought to have an inverse relationship with obesity. Several studies have demonstrated that weight loss, especially a reduction in body fat, is associated with increased circulating levels of adiponectin, particularly in its high molecular weight (HMW) form [2, 3].

Leptin levels are generally proportional to body fat mass (BFM). In the context of inflammatory conditions, particularly obesity, which is characterized by low-grade inflammation, bidirectional interactions between leptin and inflammatory processes are believed to be significant. Leptin has been shown to stimulate the production of pro-inflammatory molecules like interleukin-6 (IL-6), tumor necrosis factor-alpha (TNF-α), and IL-12, influencing immune system changes involving helper T cells and natural killer (NK) cells [4, 5].

In terms of blood cell count disturbances in obesity, studies have demonstrated a positive correlation between platelet indices—such as platelet distribution width (PDW), plateletcrit (PCT), and platelet count—and both body mass index (BMI) and the systemic immune-inflammation index (SII) [6].

Numerous studies have identified obesity as a key independent risk factor for severe COVID-19 outcomes [7–9]. The underlying pathophysiological mechanisms may involve dysfunctional AT and the persistent activation of pro-inflammatory cytokines, which are common in obesity and contribute to further metabolic dysfunction. Additionally, AT may serve as a potential viral reservoir [9, 10].

This study evaluated the relationship between adipokine levels and blood cell indices, specifically platelets, in COVID-19 patients. A secondary objective was to enhance our understanding of the endocrine functions of AT during acute viral infections like COVID-19.

## Materials and methods

### Subjects

This cross-sectional study was conducted at the University Clinical Center of Vojvodina between 2021 and 2022. A total of 76 consecutive SARS-CoV-2-positive patients of both sexes were included. The inclusion criteria were age over 18 years, a positive reverse transcription-quantitative polymerase chain reaction (RT-qPCR) test for SARS-CoV-2 from a nasopharyngeal swab, and no previously confirmed endocrinological disorders that could lead to obesity (e.g., hypothalamic or pituitary disorders, hyperprolactinemia, untreated or newly diagnosed hypothyroidism, hypercortisolism, insulinoma, and polycystic ovary syndrome). Exclusion criteria included incomplete medical records, use of medication that could alter body composition (e.g., insulin therapy, sulfonylureas, thiazolidinediones, GLP-1 agonists, glucocorticoids, lithium, antidepressants, antipsychotics, and antiepileptics), malignancies, and liver or kidney disease. Participants were divided into three subgroups based on their BMI: normal weight (BMI 18.5–24.99 kg/m^2^), overweight (BMI 25–29.99 kg/m^2^), and obese (BMI ≥ 30 kg/m^2^).

### Study protocol

The diagnosis of SARS-CoV-2 infection was confirmed by RT-qPCR testing of nasopharyngeal swabs using the commercial BGI RT-PCR assay, following the manufacturer’s instructions. Viral RNA was extracted using the Viral DNA and RNA Extraction Kit (Xi’an Tianlong Science and Technology Co., Ltd., Xi’an, Shaanxi, China) and amplified using the Gentier 96E/96R RT-PCR system (Xi’an Tianlong Science & Technology Co., Ltd., Xi’an, Shaanxi, China).

Relevant clinical data for each participant were collected from electronic medical records, including comorbidities, such as obesity, non-insulin-dependent diabetes mellitus (NIDDM) managed by diet and metformin, hyperlipoproteinemia (HLP), arterial hypertension (HTA), coronary artery disease (CAD), cardiomyopathies (CMPs), arrhythmias, and cerebrovascular disease (CVD). Each participant was assigned an identification code, and data were encrypted in the database. Data entry was verified through a double-check process.

### Anthropometric measurements

BMI was calculated for each participant using the standard formula: BMI ═ body mass (BM)/height^2^ (BH^2^). BFM was estimated using a predictive formula: BFM ═ 1.20 × BMI + 0.23 × age -- 10.8 × sex -- 5.4 (where sex: women ═ 0, men ═ 1) [11], with the results expressed as a percentage.

### Analytical procedures

Following the confirmation of a SARS-CoV-2 diagnosis and hospital admission (prior to administering any therapy specified as an exclusion criterion), all blood samples were collected according to standard local blood sampling protocols. This included collecting the samples in the morning after an overnight fasting period. A complete blood count was performed on a peripheral venous blood sample using the conventional impedance technique on a Sysmex XN instrument, manufactured by Siemens Healthcare, Germany. The remaining portion of the sample was used to separate serum, which was stored at –20 ^∘^C for subsequent analysis of serum adiponectin and leptin levels.

Serum adiponectin concentration was measured using an Adiponectin Enzyme-Linked Immunosorbent Assay (ELISA) kit from EUROIMMUN Medizinische Labordiagnostika AG in Lübeck, Germany, following the manufacturer’s instructions. The assay demonstrated linearity across the investigated concentration range (0.92–36.25 µg/mL). The intra-assay coefficients of variation (CVs) for adiponectin were 5%, 4.6%, and 7.5%, while the inter-assay CVs were 8.4%, 6.7%, and 6.6%. Serum leptin concentrations were quantified using a Leptin ELISA kit from Mediagnost, Reutlingen, Germany, also following the manufacturer’s protocol. The assay achieved a sensitivity of less than 0.01 ng/mL for leptin. Samples were diluted according to the provided guidelines based on BMI, with dilution ratios of 1:10 and 1:20. The leptin intra-assay CVs were 4.35% and 2.63%, and the inter-assay CVs were 7.2%, 6.1%, and 7.5%, all falling below 10%.

### Ethical statement

The study complied with the Declaration of Helsinki and was approved by the Ethics Committees of the University Clinical Center of Vojvodina, Novi Sad, Serbia (approval number: 00-153), and the Faculty of Medicine, Novi Sad, Serbia (approval number: 01-39/94/1). All participants provided informed consent.

### Statistical analysis

Data were analyzed using standard descriptive and inferential statistical methods via SPSS version 23 (IBM Corp., Armonk, NY, USA) and Jeffreys’s Amazing Statistics Program (JASP) version 0.18.3. The distribution of variables was assessed using the Shapiro–Wilk test. Continuous variables with asymmetric distributions were reported as medians (Me) and interquartile ranges (IQR). Categorical variables were presented as absolute numbers and percentages. The Mann–Whitney *U* test was used for non-parametric data to compare two independent samples, while the Kruskal–Wallis *H* test and post-hoc Dunn’s tests were used for comparisons among multiple independent groups. The χ^2^ (chi-square) test was applied for categorical data comparisons. Where necessary, the Bonferroni correction was applied to *P* values to adjust for multiple comparisons. Spearman’s rank correlation coefficient (rho) was used to explore relationships between variables of interest. Additionally, simple and multiple linear regression analyses were conducted to examine the association between serum leptin levels and platelet indices. The regression models were adjusted for BMI. Statistical significance was set at *P* < 0.05, and results were presented in both tabular and graphical formats.

**Table 1:** Anthropometric parameters and the levels of the studied adipokines

**Parameter**	**Normal weight** **(A)** ***n* ═ 27**	**Overweight** **(B)** ***n* ═ 24**	**Obese** **(C)** ***n* ═ 25**	****P* value***	***P* value** **A vs B**	****P* value*** **B vs C**	****P* value*** **A vs C**
**Age (years)**	65 (44–78)	55 (45–72.25)	57 (50–67)	0.711^a^	0.568^b^	0.831^b^	0.425^b^
**Gender (F/M)**	F 17 (62.96%) M 10 (37.03%)	F 8 (33.3%) M 16 (66.7%)	F 9 (36%) M 16 (64%)				0.051^c^
**BMI (kg/m^2^)**	21.5 (20.3–23.8)	27.7 (26.3–28.95)	33.1 (30.9–37.5)	<0.001^a^	<0.001^b^	<0.001^b^	<0.001^b^
**BFM (%)**	31.33 (24.17–35.58)	31.13 (27.36–41.86)	39.47 (33.88–48.81)	<0.001^a^	0.269 ^b^	0.003 ^b^	<0.001^b^
**Adiponectin** **(µg/mL)**	8.05 (5.73–11.74)	5.92 (4.4–7.78)	4.01 (3.68–6.1)	0.002^a^	0.037^b^	0.18^b^	<0.001^b^
**Leptin** **(ng/mL)**	5.60 (2.25–13)	9.75 (3.6–23.82)	21.80 (8.2–51)	0.003^a^	0.144^b^	0.059^b^	<0.001^b^
**ALR**	1.44 (0.71–4.21)	0.41 (0.25–1.24)	0.23 (0.13–0.54)	<0.001^a^	0.025^b^	0.045^b^	<0.001^b^

^a^Kruskal–Wallis test; ^b^Post-hoc Dunn’s test; ^c^Chi-square test. BMI: Body mass index; BFM: Body fat mass; ALR: Adiponectin/leptin ratio; F/M: Female/Male.

**Table 2:** Participants comorbidities

**Comorbidities**	***N***	**%**
Obesity	24	31.58
NIDDM (diet/metformin only)	3	3.95
HLP	3	3.95
HTA	41	53.95
CAD	3	3.95
CMPs	5	6.58
Arrhythmia	6	7.90
CVD	4	5.26

NIDDM: Non-insulin dependent diabetes mellitus; HLP: Hyperlipoproteinemia; HTA: Arterial hypertension; CAD: Coronary artery disease; CMPs: Cardiomyopathies; CVD: Cerebrovascular disease.

## Results

Table 1 presents the anthropometric parameters of participants and the levels of the studied adipokines. Table 2 displays the comorbidities prevalent among the study participants, such as obesity (31.58%), cardiovascular diseases (72.38%), and CVDs (5.26%). Given the role of obesity in the progression of COVID-19 and its status as a major independent risk factor for severe disease, adiponectin and leptin levels were evaluated in the serum. Obese subjects exhibited significantly higher leptin levels compared to those with normal weight (*P* < 0.001). Specifically, the obese group had a median leptin level of 21.80 ng/mL (IQR 8.2–51), while the overweight and normal-weight groups had median values of 9.75 ng/mL (IQR 3.6–23.82) and 5.60 ng/mL (IQR 2.25–13), respectively (Table 1). Post-hoc analysis showed a significant difference in leptin concentrations between the normal-weight and obese groups (A vs C, *P* < 0.001, Table 1). Conversely, obese subjects had the lowest median adiponectin levels (4.01 µg/mL, IQR 3.68–6.1) compared to the overweight (5.92 µg/mL, IQR 4.4–7.78) and normal-weight groups (8.05 µg/mL, IQR 5.73–11.74). Dunn’s test revealed a significant difference in adiponectin levels between the normal-weight and obese groups (A vs C, *P* < 0.001). However, after Bonferroni correction, no significant difference between the normal-weight and overweight groups was detected.

**Figure 1.**
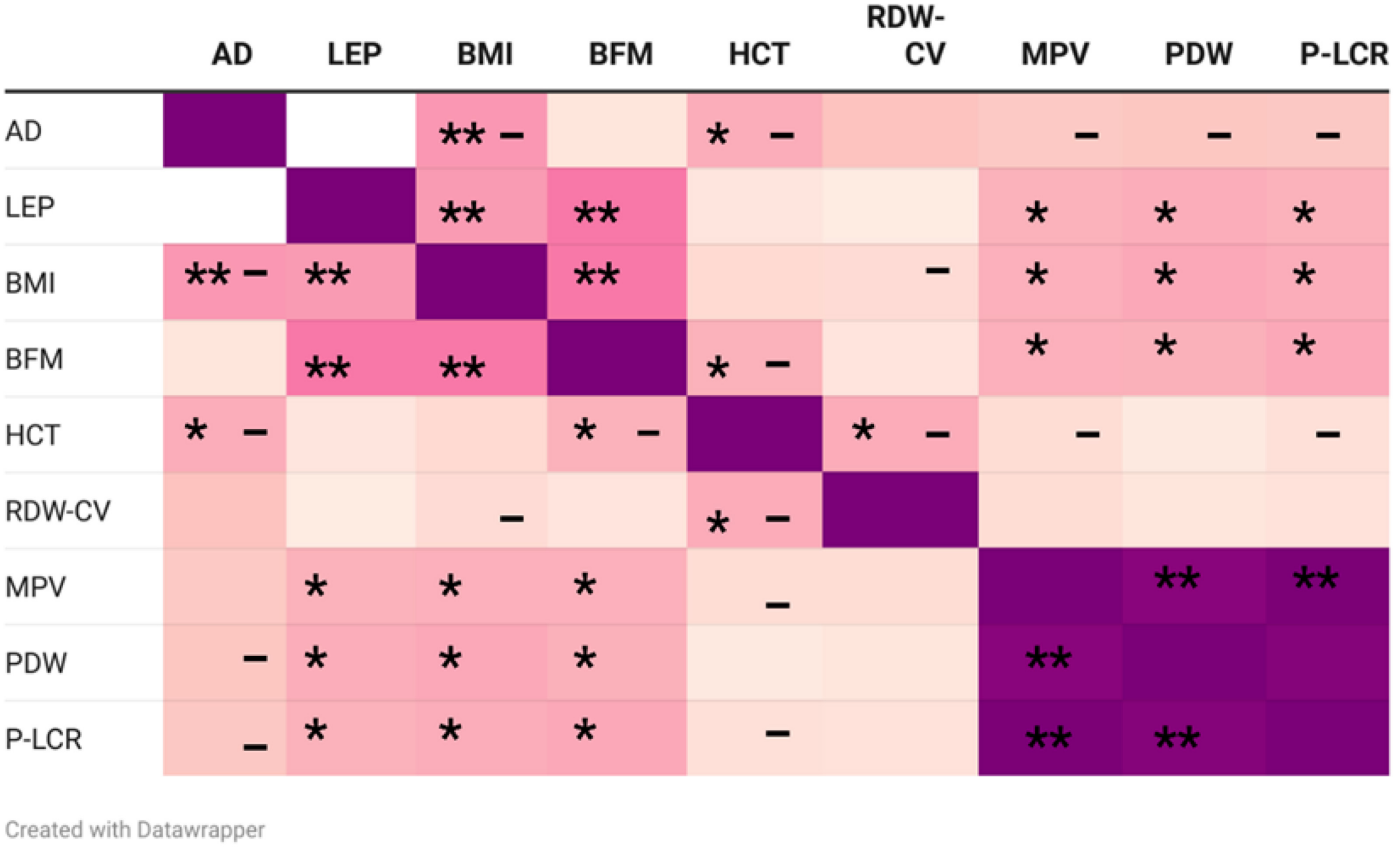
**Heatmap of the Spearman correlation (*r*) between adipokines, BMI, BFM, and blood cell indices.** AD: Adiponectin; LEP: Leptin; BMI: Body mass index; BFM: Body fat mass; HCT: Hematocrit; RDW-CV: Red cell distribution width-coefficient of variation; MPV: Mean platelet volume; PDW: Platelet distribution width; P-LCR: Platelet-large cell ratio. Spearman correlations: *P < 0.05; **P < 0.001; - negative correlation. **Photo & Design software:** Created with https://www.datawrapper.de/ and http://www.biorender.com/.

Based on the classification system proposed by Frühbeck et al. [12] (ALR > 1 indicates normal risk; ALR 0.5–1 indicates moderately elevated cardiometabolic risk; ALR < 0.5 indicates very high cardiometabolic risk), obese participants had the lowest ALR values (0.23, IQR 0.13–0.54, Table 1), placing them, along with the overweight group (0.41, IQR 0.25–1.24), in the very high cardiometabolic risk category. There was a strong negative correlation between adiponectin levels and BMI (ρ ═ –0.430, *P* < 0.001, Figure 1), and a significant positive correlation between leptin levels and both BFM (ρ ═ 0.547, *P* < 0.001, Figure 1) and BMI (ρ ═ 0.421, *P* < 0.001, Figure 1).

**Figure 2.**
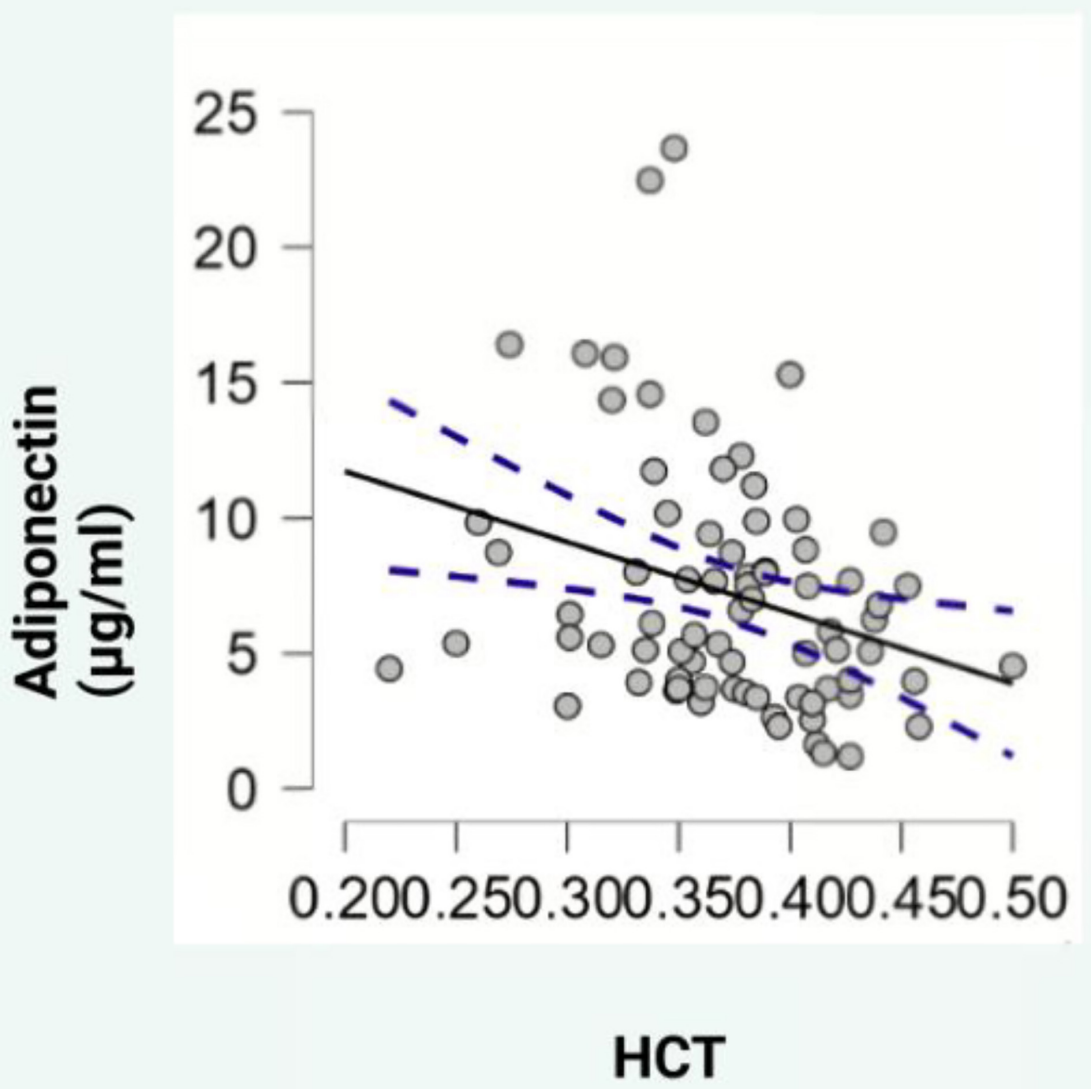
**Scatterplot depicting the correlation between adiponectin and HCT.** HCT: Hematocrit. **Photo & Design software:** Created with http://www.biorender.com/.

**Figure 3.**
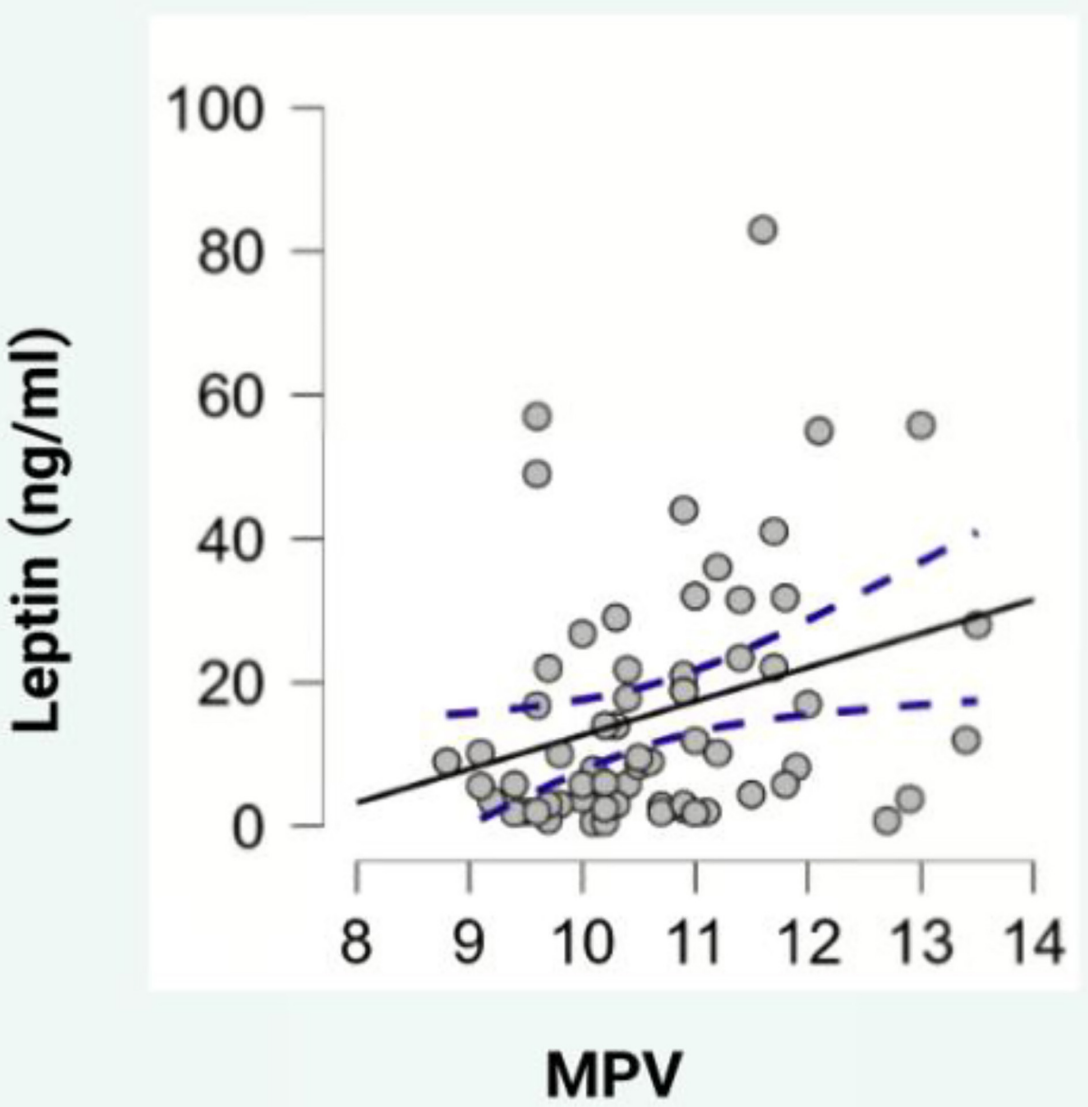
**Scatterplot depicting the correlation between leptin and MPV.** MPV: Mean platelet volume**. Photo & Design software:** Created with http://www.biorender.com/.

**Figure 4.**
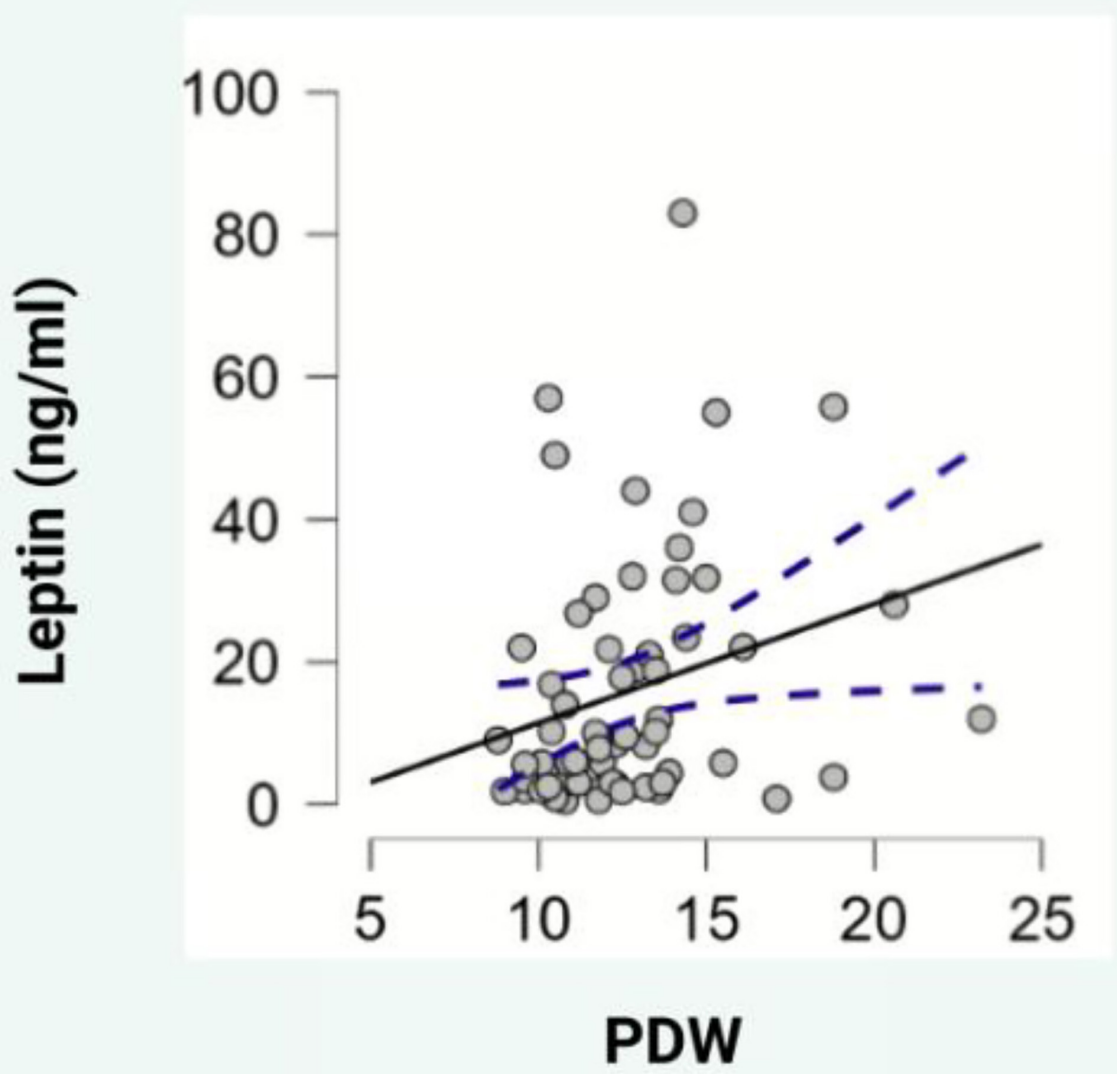
**Scatterplot depicting the correlation between leptin and PDW.** PDW: Platelet distribution width. **Photo & Design software:** Created with http://www.biorender.com/.

**Figure 5.**
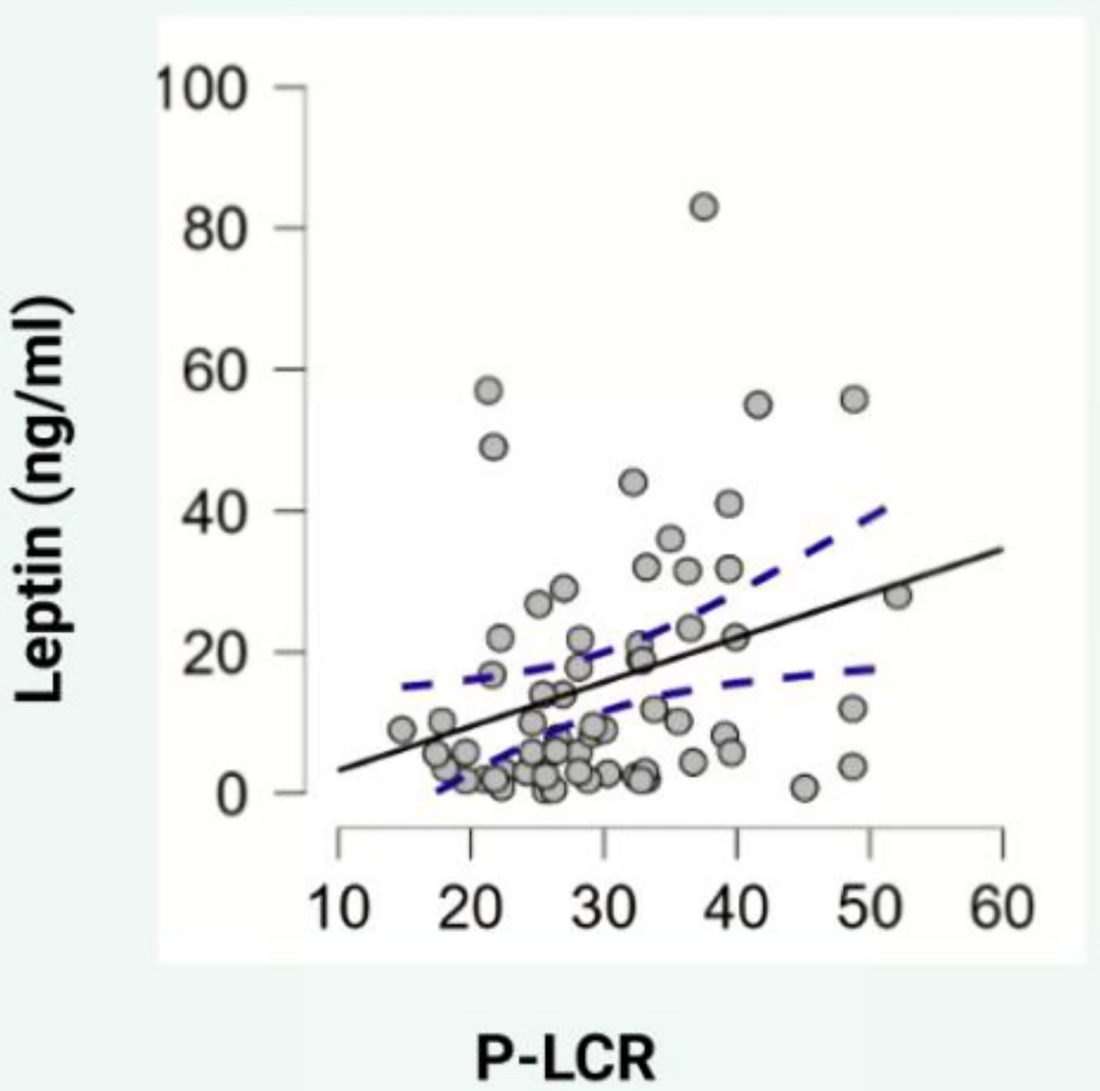
**Scatterplot depicting the correlation between leptin and P-LCR.** P-LCR: Platelet-large cell ratio. **Photo & Design software:** Created with http://www.biorender.com/.

Regarding blood parameters, adiponectin levels showed a negative correlation with hematocrit (HCT) (ρ ═ --0.329, *P* < 0.05, Figure 2), while leptin levels exhibited positive correlations with mean platelet volume (MPV) (ρ ═ 0.307, *P* < 0.05, Figure 3), PDW (ρ ═ 0.325, *P* < 0.05, Figure 4), and platelet-large cell ratio (P-LCR) (ρ ═ 0.305, *P* < 0.05, Figure 5). Simple regression analysis (Tables 3–5) revealed higher beta coefficients for leptin (MPV: β ═ 0.295, *P* < 0.05; PDW: β ═ 0.264, *P* < 0.05; P-LCR: β ═ 0.305; *P* < 0.05) than for BMI (MPV: β ═ 0.274, *P* < 0.05; PDW: β ═ 0.261, *P* < 0.05; P-LCR: β ═ 0.295; *P* < 0.05). Multiple linear regression (Tables 3–5) was used to further investigate the relationship between MPV, PDW, and P-LCR as dependent variables, and leptin and BMI in three independent models. No significant multicollinearity issues were detected in the preliminary analyses.

Significant models (*P* < 0.05) explained 9% of the MPV variance, 7.1% of the PDW variance, and 10.3% of the P-LCR variance. When examining the independent variables across all three models, serum leptin levels exhibited a slightly higher beta coefficient compared to BMI, though the significance level was low (*P* < 0.1) for MPV and P-LCR. BMI did not reach statistical significance.

The results are presented in Table 6, detailing the adipokine values investigated in relation to hospital unit type. Although patients in the medical ward showed numerically higher levels of adiponectin (6.69 µg/mL, IQR 3.70–9.43) and ALR (0.72, IQR 0.35–1.89) and numerically lower levels of leptin (8.00 ng/mL, IQR 3.00–21.20) compared to patients in the semi-intensive care unit (SICU) and intensive care unit (ICU) (adiponectin 5.23 µg/mL, IQR 4.33–7.94; ALR 0.32, IQR 0.21–0.90; leptin 16.40 ng/mL, IQR 4.3–34.50), these differences did not reach statistical significance.

Furthermore, no statistically significant association was found between adiponectin (ρ ═ –0.054, *P* ═ 0.642) or leptin levels (ρ ═ 0.192, *P* ═ 0.097) and the type of hospital setting, which correlated with the severity of COVID-19.

## Discussion

Red blood cells (RBCs), the most abundant human cells, and platelets orchestrate the primary immune response against SA-binding pathogens, including SARS-CoV-2. SA residues, heavily expressed in these blood cells, mediate cell interaction and subsequent vascular morbidities [13–15]. After this initial virus-host contact, RBC aggregates form and undergo phagocytosis, which in severe COVID-19 cases exceeds the described sequestration capacity [14]. Furthermore, endothelial injury and RBC aggregation can trigger a coagulation cascade, leading to thrombosis [13, 16, 17]. Reduced HCT levels [18] and an accelerated erythrocyte sedimentation rate (ESR) [19] are indicators of RBC clumping.

Our study shows that adiponectin levels negatively correlate with HCT. Hindsberger et al. [20] reported that higher adiponectin levels upon hospital admission are associated with reduced mortality rates. In our subgroup analysis, obese individuals had the lowest median adiponectin levels. Additionally, it is well-established that adiponectin inhibits IL-6 expression in murine models [21] and human bronchial epithelial cells [22], leading to reduced inflammatory reactions. This suggests that the low adiponectin levels often observed in obese patients may contribute to the development of more severe forms of COVID-19 [23].

**Table 3:** Simple and multiple linear regression—MPV

**Model**	**Model *P* value**	**Adjusted** **r^2^**	**DW**	**Dependent variable**	**Independent variable (s)**	**β**	***P***	**VIF**	**Tol**
**1.**	***<0.05***	0.072	1.671	MPV	Leptin	0.295	***<0.05***	**/**	**/**
**2.**	***<0.05***	0.060	1.665	MPV	BMI	0.274	***<0.05***	**/**	**/**
**3.**	***<0.05***	0.089	2.350	MPV	Leptin BMI	0.223 0.192	***0.090** 0.142*	1.153 1.153	0.867 0.867

BMI: Body mass index; MPV: Mean platelet volume; VIF: Variance inflation factor; Tol: Tolerance values; DW: Durbin–Watson.

**Table 4:** Simple and multiple linear regression—PDW

**Model**	**Model *P* value**	**Adjusted** **r^2^**	**DW**	**Dependent variable**	**Independent variable (s)**	**β**	***P***	**VIF**	**Tol**
**1.**	***<0.05***	0.055	1.601	PDW	Leptin	0.264	***<0.05***	**/**	**/**
**2.**	***<0.05***	0.053	1.686	PDW	BMI	0.261	***<0.05***	**/**	**/**
**3.**	***<0.05***	0.071	2.316	PDW	Leptin BMI	0.195 0.189	*0.143 0.157*	1.154 1.154	0.867 0.867

BMI: Body mass index; PDW: Platelet distribution width; VIF: Variance inflation factor; Tol: Tolerance values; DW: Durbin–Watson.

**Table 5:** Simple and multiple linear regression—P-LCR

**Model**	**Model *P* value**	**Adjusted** **r^2^**	**DW**	**Dependent variable**	**Independent variable (s)**	**β**	***P***	**VIF**	**Tol**
**1.**	***<0.05***	0.078	1.723	P-LCR	Leptin	0.305	***<0.05***	**/**	**/**
**2.**	***<0.05***	0.072	1.708	P-LCR	BMI	0.295	***<0.05***	**/**	**/**
**3.**	***<0.05***	0.103	2.387	P-LCR	Leptin BMI	0.227 0.212	***0.084** 0.106*	1.154 1.154	0.867 0.867

BMI: Body mass index; P-LCR: Platelet-large cell ratio; VIF: Variance inflation factor; Tol: Tolerance values; DW: Durbin–Watson.

**Table 6:** Hospital setting and the levels of the studied adipokines

**Parameter**	**Medical ward** **(A)** ***n* ═** **52**	**SICU/ICU** **(B)** ***n* ═ 24**	****P* value***
**Adiponectin** **(µg/mL)**	6.69 (3.70–9.43)	5.23 (4.33–7.94)	0.643^a^
**Leptin** **(ng/mL)**	8.00 (3.00–21.20)	16.40 (4.30–34.50)	0.098^a^
**ALR**	0.72 (0.35–1.89)	0.32 (0.21–0.90)	0.077^a^

^a^Mann–Whitney *U* test. SICU: Semi-intensive care unit; ICU: Intensive care unit; ALR: Adiponectin/leptin ratio.

Flikweert et al. [23] demonstrated that critically ill SARS-CoV-2 patients had lower adiponectin levels (survivors: 2.4 µg/mL, IQR: 1.5–4.2; non-survivors: 3.0 µg/mL, IQR: 1.7–6.9), aligning with our study’s findings.

Additionally, we discovered that critically ill COVID-19 patients hospitalized in the ICU had lower serum adiponectin concentrations compared to those hospitalized in medical wards, although this difference was not statistically significant. Kearns et al. [24] demonstrated that adiponectin levels, measured at three time points (0, 24, and 72 h of hospitalization), were significantly lower in patients with respiratory failure due to COVID-19 compared to those with respiratory failure associated with other infectious diseases. Even after accounting for factors, such as BMI, age, and gender, this difference remained statistically significant.

Moreover, Perrotta et al. [25] discovered that adiponectin levels in COVID-19 patients were elevated but still much lower than those in healthy individuals. Additionally, they found that HMW oligomers, the most physiologically active forms of adiponectin, positively correlated with lung ultrasound (LUS) scores [25]. In our study, obese patients had the lowest numerical values of the adiponectin/leptin ratio (ALR), placing them in the “very high cardiometabolic risk” category, as classified by Frühbeck et al. [26]. This category also included patients admitted to the ICU.

However, Di Filippo et al. [27] presented the ALR in their study as a measure of a regulatory defensive mechanism that mitigates damage related to systemic inflammation, supporting their findings that patients with milder disease had the lowest ALR. While the protective effects of adiponectin on cardiometabolism are widely acknowledged, it is important to consider the adiponectin paradox [28, 29]. This paradox, particularly as individuals age and develop chronic conditions, suggests that elevated adiponectin levels may be associated with the onset of chronic illnesses and increased mortality from diabetes mellitus [30]. In our study, we found that normal-weight individuals had the highest median age and adiponectin levels, which were significantly higher than those of obese patients.

Mineo et al. [31] revealed that patients with severe COVID-19 had significantly higher adiponectin levels compared to those with non-severe cases. Additionally, it is crucial to consider the timing of blood sampling for laboratory analysis. For instance, in our study, blood samples were collected shortly after the patient’s admission.

Our findings also showed a positive correlation between MPV and leptin levels. A simple regression analysis, including MPV, revealed that leptin had a higher beta coefficient than BMI. However, when a multiple linear regression was conducted, incorporating leptin and BMI, the model remained statistically significant, though leptin’s beta coefficient remained larger than BMI’s, despite its low significance level (*P* < 0.1). Conversely, BMI did not achieve statistical significance.

According to current scientific understanding, elevated MPV may indicate increased platelet activation, production, and aggregation [32]. Larger platelets, due to their greater enzymatic and metabolic activity, have been associated with higher prothrombotic potential [33], demonstrated by increased thromboxane A2 (TXA2) levels, enhanced granular content, and increased expression of adhesive proteins (GpIIb/IIIa and GpIb) [34]. Studies have shown a positive correlation between MPV and various cardiometabolic conditions, including dyslipidemia, obesity, insulin resistance, metabolic syndrome, and hypertension [32]. The endocrine function of AT in relation to insulin resistance—particularly through adipocytokines such as adiponectin and leptin, along with various interleukins and nitric oxide (NO) alterations—may drive megakaryocytes to produce larger platelets [35]. Furthermore, hyperglycemia, a common marker of diabetes mellitus and insulin resistance, can induce osmotic swelling in platelets, raising MPV [36].

Our study also demonstrated a positive correlation between leptin, PDW, and P-LCR. Simple regression analyses incorporating PDW, P-LCR, leptin, and BMI indicated that leptin’s beta coefficients were slightly higher than BMI’s. However, in a multiple regression analysis, leptin levels still showed slightly higher beta coefficients compared to BMI. The significance level for P-LCR was *P* < 0.1, while BMI did not reach statistical significance. PDW reflects platelet function, activation, and destruction, as studies have found a positive correlation between PDW and diseases linked to endothelial dysfunction [37, 38]. Upon activation, platelets change shape from discoid to spherical, altering PDW [32].

A connection between hyperleptinemia, hypoadiponectinemia, and PDW has also been documented [39]. More research is needed to further examine the relationship between P-LCR, a newer platelet parameter, and various diseases. However, a study by Chen et al. [38] demonstrated a positive correlation between aging markers and platelet characteristics, including MPV, PDW, and P-LCR, shedding light on its potential pathobiology. Platelets are thought to contribute to inflammation by releasing pro-inflammatory mediators and attracting leukocytes, which may cause telomere shortening and endothelial dysfunction through a reactive oxygen species (ROS)-dependent mechanism [38]. Leptin levels were lower in patients from the medical ward compared to those in the ICU or SICU. Earlier research by Wang et al. [40] demonstrated that obese patients had higher leptin levels, which may contribute to systemic inflammation and monocyte activation, making them more susceptible to severe forms of COVID-19. Similar findings were reported by Van der Voort [41] and Perrota et al. [25]. However, our study did not find a significant correlation between leptin levels and COVID-19 severity. We attribute this discrepancy to the findings of Perrota et al. [25], who classified leptin as a minor predictor of COVID-19 severity, likely due to heterogeneity among patient groups in different studies.

Additionally, it is important to consider that pro-inflammatory adipocytokines may increase or decrease more slowly in obese individuals compared to those with normal weight, due to decreased activity of NK cells and dysregulation in CD4+ and CD8+ T cells. This could lead to a delayed immune response and a prolonged hyperinflammatory state in obese COVID-19 patients, influencing adipokine status based on the timing of the investigation [42].

Our study has several limitations. The cross-sectional design and small sample size limit the ability to determine causal relationships between the factors studied. Furthermore, we could not perform a comprehensive survival analysis, so these findings need validation in larger cohorts, accounting for age and gender differences. Limited resources also prevented the inclusion of an appropriate control group. Additionally, laboratory data were assessed at a single time point. To estimate BFM, we used a formula from the literature, as isolation measures during the COVID-19 pandemic restricted access to more precise imaging methods. This formula should be interpreted cautiously, particularly in obese individuals, where it may slightly overestimate BFM. Nevertheless, to our knowledge, our study is the first to examine AT endocrine function in response to SARS-CoV-2 infection in the Republic of Serbia. Our investigation offers an additional perspective on the complex relationship between COVID-19 and AT.

## Conclusion

The study’s findings indicate a negative association between HCT and adiponectin levels. In contrast, a positive association was observed between leptin levels and MPV and P-LCR, even after controlling for BMI. Patients in the SICU/ICU had higher leptin levels but lower adiponectin and ALR levels compared to those in conventional medical wards. Additionally, the obese group exhibited the highest leptin levels and the lowest ALR, indicating a significant cardiometabolic risk. Our findings may provide further insights into AT’s role in the complex cascade involving COVID-19 pathogenesis and platelet activation.

## Data Availability

The data associated with the manuscript are available from the corresponding author upon reasonable request.
